# Measuring Social Networks for Medical Research in Lower-Income Settings

**DOI:** 10.1371/journal.pone.0105161

**Published:** 2014-08-25

**Authors:** Laura Kelly, Shivani A. Patel, K. M. Venkat Narayan, Dorairaj Prabhakaran, Solveig A. Cunningham

**Affiliations:** 1 Graduate Group in Demography, University of Pennsylvania, Philadelphia, Pennsylvania, United States of America; 2 Hubert Department of Global Health, Emory University, Atlanta, Georgia, United States of America; 3 Center for Chronic Disease Control, New Delhi, India; 4 Chronic Disease Epidemiology, Public Health Foundation of India, New Delhi, India; Kenya Medical Research Institute - Wellcome Trust Research Programme, Kenya

## Abstract

Social networks are believed to affect health-related behaviors and health. Data to examine the links between social relationships and health in low- and middle-income country settings are limited. We provide guidance for introducing an instrument to collect social network data as part of epidemiological surveys, drawing on experience in urban India. We describe development and fielding of an instrument to collect social network information relevant to health behaviors among adults participating in a large, population-based study of non-communicable diseases in Delhi, India. We discuss basic characteristics of social networks relevant to health including network size, health behaviors of network partners (i.e., network exposures), network homogeneity, network diversity, strength of ties, and multiplexity. Data on these characteristics can be collected using a short instrument of 11 items asked about up to 5 network members and 3 items about the network generally, administered in approximately 20 minutes. We found high willingness to respond to questions about social networks (97% response). Respondents identified an average of 3.8 network members, most often relatives (80% of network ties), particularly blood relationships. Ninety-one percent of respondents reported that their primary contacts for discussing health concerns were relatives. Among all listed ties, 91% of most frequent snack partners and 64% of exercise partners in the last two weeks were relatives. These results demonstrate that family relationships are the crux of social networks in some settings, including among adults in urban India. Collecting basic information about social networks can be feasibly and effectively done within ongoing epidemiological studies.

## Introduction

Social relationships pattern daily life and may offer powerful leverages for health interventions. The constellation of social relationships we possess is our *social network*. Social network analysis emerged in anthropology and sociology as a method to study social structure [1], [2]. A fundamental feature of social network analysis is the focus on social connections among individuals and the patterns and consequences of these connections. Individuals and their actions are embedded within social relationships, and social networks are likely to be important determinants of health. It is imperative for health researchers and practitioners to consider individuals' social networks and their significance for both individual and population health.

A growing body of literature has drawn on network analysis to study how the social environment may influence health. Social networks can affect the flow of ideas and resources that shape opportunities and constraints [2]. Berkman and colleagues proposed that social networks shape individual health and health behaviors primarily through (1) social support (e.g., sharing of information, and financial and emotional assistance); (2) social influence (e.g., comparison with peers, pressure from norms and peers, and imitation); (3) social participation and engagement (e.g., attending functions and bonding with friends); (4) person-to-person contact (e.g., physical exposure to infectious agent); and (5) access to resources and material goods (e.g., jobs, healthcare, housing) [2], [3].

Studies of health in low- and middle-income (LMIC) settings face multiple challenges, including limited infrastructure and institutional resources to conduct research. The inclusion of social network measurements poses additional challenges, requiring attention to additional concepts and measurements external to the direct scope of traditional public health research. Yet social network analyses may be particularly useful for understanding health behaviors in LMIC settings, where social ties are often the primary resource for acquiring health information and material resources due to the absence of more formal markets (Lomnitz 1977). Resource transfer through personal networks may be most important in environments with limited institutional, state-based and private-sector support.

Despite evidence for the importance of networks on health, social network analyses in health research have been limited, especially in LMIC settings. One reason may be the limited guidance available to health researchers regarding how information about social networks and their influences on health can be collected and analyzed. In this report, we provide basic guidance by describing how we developed and administered a survey for measuring social networks and health within a large epidemiologic surveillance study in urban India. We describe the instrument and preliminary data from this project.

### Social network concepts and their application to health

Table 1 presents a basic vocabulary for social network analysis. Social networks consist of relationships (*ties*) between individuals, groups, or organizations (*actors*). Most methods for collecting data on social networks can be described as egocentric or sociometric. The *egocentric* approach, which generates personal network data, measures a social network from the perspective of a focal index respondent (*ego*). The ego nominates a list of his or her social relations (*network members* or *alters*), often the ones who are most important to him or her. Egocentric data may be most important when the ego's perception of his or her social relations, for example their beliefs and behaviors, are of interest to the researcher [4]. In the *sociometric* approach, data are collected directly from each member of a network within a distinct, bounded community such as a school, a place of employment, or a club. Sociometric network data are most useful if objective characteristics of each member of a network are of primary interest, rather than subjective information reported by an ego.

**Table 1:** Social network vocabulary.

Network terms	Definition
Actor	Network members, either distinct individuals or collective units
Ego	Focal actor
Alter	Actors connected to the ego through ties
Tie	Relationship between actors within a network
Egocentric	Personal network defined from a focal actor's (ego) perspective
Sociometric	Complete network of ties among a bounded community of actors
Name generator	Prompt to collect nomination of actors (alters) related to the focal ego
Name interpretation	Question designed to elicit information from ego about nominated alters, characterizes each tie
Network size	Number of nominated ties among all possible ties
Network exposure	The number of ties within the personal network exhibiting a specific attribute not shared by the ego among all nominated ties
Network homogeneity	The number of ties within the personal network exhibiting an attribute shared by the ego among all nominated ties
Tie diversity	The number of distinct ties reported among all nominated ties, in terms of relationship type such as kin vs. friend
Tie mutliplexity	The variety of distinct roles or resources among each tie, such as drinking partner vs. exercise partner

When deciding which approach to take, researchers must consider both their study objectives and feasibility. Egocentric measurement is a practical option for exploratory studies because it relies exclusively on the focal respondent, without the major feat of finding and enrolling his or her network members. This method allows researchers to learn about social networks in large unrestricted populations [5]. Egocentric data can be collected from individual study participants using traditional health research methods, namely interviews, self-completed surveys, or direct observation. Concerns of validity and reliability inherent in respondent bias are therefore consistent with traditional data collection methods. Egocentric network attributes can be treated as “exposures” in health studies and are possible to analyze using conventional analytic software such as SAS or STATA. Sociometric instruments require that all members of a network be interviewed, which requires all network members to be pre-identified, such as from school or company rosters; a process that can pose major logistical challenges [6]. Sociometric network analysis can be used to map out entire networks with specialized software such as Pajek, NodeXL, Gephi, NetMiner, NetworkX, or iGraph. Compared to sociometric network data, egocentric network data generally incur a lower researcher cost, reduce respondent burden as respondents self-nominate alters rather than providing information on an entire roster of individuals in a given community, and are free from organizational or geographical limitations [4], [6], [7]. We focus here on egocentric instruments due to their high feasibility for health researchers and easy integration into existing data collection practices.

Network studies ask respondents about particular types of network members such as friends, partners, or individuals who provide them with specific types of support. Network data collection typically begins by asking each respondent to provide the names of individuals who fall within the type of social relations of interest to the study. This question, called a *name generator,* sets the scope of subsequent questions about the network. For example, the focal respondent may be asked to name only alters with whom he or she discusses important matters [8]. Once names are listed, they are used to aid in asking follow-up questions (*name interpretation*) designed to assess substantive attributes of each tie such as type of relation (e.g., family, friend, coworker), strength of the relationship and frequency of contact (measures of closeness), demographic and health characteristics, and shared activities and beliefs.

From these egocentric network attributes, researchers can construct measures that are commonly used in analysis: network size, network exposure, network homogeneity, and network diversity.

*Network size*, the number of alters in a person's social network, is a measure of the structure of the network [4]. In practice, the number of named network members is generally capped at a maximum (often 5 or 10) to limit survey length, as each named member requires follow-up questions. Network size has been shown to be important for health. Smaller social networks are associated with poorer health including susceptibility to rhinovirus contact [9], stroke events [10], depression scores [11], lower self-esteem and quality of life [12], and mortality [13]–[16]. Smaller networks may reflect social isolation, which in turn can negatively affect health because it entails low social engagement, social support and access to material resources [17].

*Network exposure* indicates the characteristics to which the respondent is exposed through his or her relationships in terms of specific attributes of interest to the study, such as ethnicity or gender, or in terms of specific behaviors, such as smoking or drinking alcohol. Questions about the attribute or behavior are asked with respect to each named network member to quantify network exposure. Network exposure to a particular characteristic is a fundamental measure of network influence. Network influence, a central tenet of social network analysis, presumes the diffusion of attributes throughout a network via social contacts. Diffusion is most evident in the spread of infectious disease within a social network; for example, the number of ego's sexual partners who are infected with syphilis [18] or HIV [19]–[21] are predictive of the ego's risk of contracting the disease. Similarly, social epidemiologists provide empirical evidence of “contagion” of health behaviors within networks such as adoption of risky sexual practices as well as drug, tobacco and alcohol use [22]–[30]. There is also growing evidence that exposure to suicide events [31], [32], obesity and physical activity [33]–[39], academic success [40], loneliness [41], happiness [42] and sleeplessness [43] via social network ties increases the spread of each event or disease state to other ties in the network.

A complementary but distinct network measure is *network homogeneity*, which measures the extent of similarity within the respondent's network. Based on questions asked about each relation, network homogeneity is calculated as the proportion of alters in one's network exhibiting the same attribute as the respondent. For example, the proportion of alters living in the same city as the respondent or the proportion of alters of the same gender as the respondent may be relevant to the study. *Network homogeneity* provides a measure of the extent to which an ego is similar to his or her alters. Any attribute of interest can be assessed in terms of network homogeneity including demographic, socioeconomic, attitudinal, or behavior characteristics. Higher network homogeneity may facilitate exchange of information, diffusion of innovation [44] or provision of social support [45].

Information on the social relationship itself, or the dyadic tie, requires additional indicators that may be useful to health researchers. Characteristics of each distinct tie that are commonly analyzed are strength and multiplexity of the relationship. A collective measure of all ties in a given network is network diversity.

Tie *strength* is a measure of closeness operationalized as the level of intimacy or frequency of contact between the respondent and his or her network members. In egocentric data, relative strength of network ties can be assessed by a ranking of alters in the name generator prompt, e.g. “please list five friends *in order of closeness*,” or determining the frequency of contact in the previous two weeks. Similar to network size, measures of *tie strength* provide information about social integration. For example, adolescents reporting higher closeness with network members report fewer sexual partners [22]. HIV has been shown to be concentrated among those whose close social contacts are injection drug users, with social distance from such individuals reducing viral spread [24]. Individuals with stronger social relationships had up to a 50% reduction in mortality risk [46].

*Network mutiplexity* measures the diversity of roles or resources, influenced by the research question, which exists for each tie between the ego and network member. Measures of network multiplexity quantify the variety of resources, including behavioral, attitudinal, or material, flowing between each dyad and can be assessed by asking about specific activities in which the respondent engages with each alter. Multiplexity within a tie corresponds to multiple distinct functions of the social relationship, such as exercise partner, confidant, and smoking companion. In personal networks, higher network multiplexity across various domains correlates with higher likelihood of joint behavior for an outcome of interest. For example, higher network muliplexity among adolescent friends, operationalized as joint participation in athletics, homework, and school clubs, is associated with a lower likelihood of cigarette smoking for both the ego and alter [47].

*Network diversity* quantifies the number of different tie types reported in an ego's personal network such as kin, friendship, or residential ties. Higher *network diversity* tends to be associated with more favorable health outcomes. Persons with more diverse networks exhibit lower mortality risk [48], higher survival following a stroke [17], lower ischemic heart disease risk [48], and less susceptibility to rhinovirus exposure [9].

### Designing a survey to measure social networks and health

Building on a large on-going population-based study of adults in Delhi, India, we developed the Social Network Analysis Project (SNAP) instrument to quantify the associations between social networks and cardio-metabolic health. The survey was nested within the ongoing CardiometAbolic Risk Reduction in South Asia (CARRS) surveillance study, designed to monitor cardio-metabolic diseases and associated risk factors in the National Capital Territory of Delhi, India (Nair et al., 2012b).

As an add-on to an already lengthy instrument, SNAP focused on (1) a concise social network collection and (2) items relevant to our research area, cardiometabolic health. We drew on social network instruments used elsewhere including the National Longitudinal Study of Adolescent Health [49], the Malawi Diffusion and Ideational Change Project [50] and the General Social Survey [8]. The final instrument is shown in Table S1.

Table 2 displays the components of SNAP. The network survey begins with the *name generator*: “I would like to ask you a few questions about the closest people in your life. Please think of people with whom you may discuss problems or with whom you would exchange advice.” By design, respondents can nominate up to 5 network members. Eleven items collect information for each nominated network member (Items S2, S3, S4, S5, S9, S11, S12, S13, S14, S15, and S16 in the SNAP instrument found in the Material S1). Each item question is read for the first-nominated friend (designated person [A] in the instrument) using the name given by the respondent. For the remaining network members (up to 4 additional names) the questions are asked as “How about ___?” using the names of each nominated alter. As such, the interviewer collected information for each alter in turn. Three additional questions collect information on the network generally, asking respondents to choose one network member (Items S6, S7, and S8 in the SNAP instrument) based on a given set of criteria. Interviews took approximately 20 minutes to complete.

**Table 2:** Survey domains and underlying network concept.

Network Survey Domain	Item	Network construct/concept
Name generator prompt	*" I would like to ask you a few questions about the closest people in your life. Please think of people with whom you may discuss problems or with whom you would exchange advice. I will ask you for their names to keep track of them for the following questions, but the names will be kept confidential and will not be made available to anyone except the investigators of the study."*	
Name nomination in order of closeness	Could you please tell me the names of the 5 people whom you consider to be closest to you? They can be family members, friends, acquaintances, or coworkers. Please begin with the person you are closest to.	Network size/Tie strength
Name interpretation	Is ___ male or female?	Network homogeneity
	What is _____'s relationship to you?	
	Where does ____ live in relation to you?	
Health communication among network members	Of the 5 people listed, whom would you be most likely to contact if you had a health emergency or if you needed help?	Social support
	Of the 5 people listed, whom would you be most likely to speak with about a health problem?	
	Of the 5 people listed, whom would you be most likely to contact if were feeling overwhelmed or anxious?	
	How often do you speak to ______ about your own health?	Tie strength
	How often do you speak to ______ about _____'s health?	
Shared activities with network members	*"Now I'm going to ask you some additional questions about activities you have done in the past fourteen days with the people you listed."*	Network multiplexity
	Have you communicated with ____ in person, by phone, SMS, or email in the past fourteen days?	
	In the past fourteen days, how many days have you shared snacks with _____?	
	In the past fourteen days, how many days have you shared meals with _____?	
	During the past fourteen days, have you exercised, done yoga, jogged, or gone to the gym with the purpose of maintaining or improving your health with _____?	
	During the past fourteen days, have you walked or performed small tasks outside of the home, such as walking to the store with ____?	
	During the past fourteen days, have you prepared a meal together or gone grocery shopping with ____?	
Influence of network members on health	During the past fourteen days, have you used tobacco in any form (smoking, chewing, snuff, etc.) with ____?	
Network member tobacco use	To the best of your knowledge, does ____ use any form of tobacco (smoking, chewing, snuff, etc.)?	Network exposures/ Network homogeneity
Network members weight	How would you describe _____'s weight compared to your own weight?	

For each respondent, personal network size is measured as the number of reported relations out of 5 possible nominations. Network diversity is measured in terms of the number of unique tie types, for example the prevalence of relative ties or friendship ties. Network homogeneity is measured as the similarity between the ego and his or her alters for a given attribute. Network exposures to specific attributes are calculated as the proportion of reported network members exhibiting an attribute out of all reported network members.

Tie strength can be measured in multiple ways. The relative closeness of a tie within each personal network is measured by name ordering in the name generator. Respondents rank social relations according to the prompt: “Please tell me the names of the 5 people whom you consider to be closest to you. They can be family members, friends, acquaintances, or coworkers. *Please begin with the person you are closest to.*” A second measure relates to intimacy and is based on respondents' ranking of whom they would talk with about health and personal matters. Respondents are asked: “Of the people listed whom would you be most likely to contact if you had a health emergency or if you needed help?” A third measure is frequency of contact, based on respondents' answers about the frequency of sharing activities with each named alter in the previous fourteen days.

Finally, tie multiplexicity is measured through questions regarding various interactions, including discussion of health matter or shared health-related behaviors, between the respondent and each alter. While our instrument emphasized cardiometabolic behaviors to meet our broader objectives, the instrument could be adapted to address other components of health.

### Adapting the survey for the urban Indian cultural setting

We finalized wording of survey items through item-by-item consultation with local experts and through pre-testing of a convenience sample administered by local personnel, including the team of 9 CARRS fieldworkers. We modified the name generator, nomination, and interpretation items found in the existing literature to suit an urban Indian population. Pre-testing revealed that the name generator and nomination process was occasionally challenging for participants to comprehend. Several respondents listed names of children, deities, and deceased persons as social relations. In such cases, interviewers felt the need to supplement the script with explanations of what types of relationships were of interest to the research team and included unscripted prompts to consider who the participant may lean on during times of health problems or health emergencies. For name generation and nomination in the final SNAP instrument, the script to “think of people with whom you discuss problems or with whom you would exchange advice” who could be “family members, friends, acquaintances, or coworkers” was ultimately used to address this population-specific tendency of respondents.

We adapted items of shared activity to accurately measure network multiplexity. In Western settings, exercise with friends has been shown to correlate with higher frequency of exercise and is an established measure of social influence on health behaviors. Urban Indians often do not perform physical activity for the purpose of exercising to maintain health. In order to capture indicators of shared physical activity among social ties for this urban Indian population, we asked specifically about yoga, walking to the store, or performing small tasks outside of the home. Adaptations of this instrument in other settings must consider local interpretation of exercise and physical activity. It is further rare to consume food alone in India. Urban Indians normatively observe and break fasts socially, consume specific foods communally during festivals, and share food as a component of both home and work life. Both because of the social importance of food in India and our interest in cardio-metabolic health, we asked questions regarding sharing snacks and meals. Other minor considerations included asking about all forms of tobacco use, rather than only smoking, due to the high prevalence of chewing tobacco in India.

### Fielding the survey in an urban Indian setting

The CARRS parent study sampled 4,425 individuals (response rate = 96%) in 2010–2011 using a multistage cluster strategy designed to obtain a representative cohort of the New Delhi population aged 20 years and older excluding pregnant women and bed-ridden individuals. One male and one female permanent resident of each sampled household were selected for enrollment using the Kish method. The social network sub-study made no alterations to existing CARRS sampling. All 215 newly enrolling CARRS participants between May and October 2011 were invited to participate in SNAP sub-study. Of these, 208 consented to participate, resulting in a 97% response rate for SNAP. Based on interviewer feedback, only one refusal stemmed from a reluctance to share information of social contacts, with the other refusals resulting from the respondent being unable to delay leaving for work. This population exhibited high willingness to share information on social ties.

Interviewers were trained and practiced in pairs. As part of CARRS, interviewer pairs conducted household visits with household respondents interviewed in separate rooms if possible. Supervisors and the lead author continually monitored data collection: they reviewed forms each day for discrepancies and item non-response. When possible problems were identified, supervisors and the interviewer worked together to examine and correct the issue. Interviewers were retrained at monthly meetings or as needed according to field supervisors' assessments. SNAP data were entered and managed by the first author in a Microsoft Access database. The database was checked daily against the paper survey to identify data entry errors.

During implementation in the field, interviewers were rarely able to conduct the interviews in private. Previous research in India also observed the communal nature of interviews conducted in homes [51]. We addressed this challenge by adjusting the interviewer comments question at the end of the survey and specified that the interviewer record whether anyone other than the respondent was present during the SNAP interview. About 87% of interviews were conducted with at least one other person present.

Many longitudinal surveys, including CARRS, collect contact information such as name, relationship to respondent (neighbor, relative, friend, employer, or other), address, and telephone number, for additional individuals who could help locate the respondent if needed for the next wave of data collection. CARRS asked respondents to provide such information for two contacts. A follow-on project could be established to directly collect information from these relations, who are likely to be among the closest relations of the respondent. Such data allows a partially sociometric study that would validate ego-reported alter attributes.

#### Ethical Statement

The CARRS surveillance study was reviewed and approved by the Institutional Ethics Committee of the Public Health Foundation of India in accordance with the Indian Council for Medical Research and the Emory University Institutional Review Board in accordance with Federal and Institutional criteria. SNAP was approved as an amendment to the CARRS surveillance study by the Institutional Ethics Committee of the Public Health Foundation of India and the Emory University Institutional Review Board. All participants of this project provided voluntary informed written consent according to the procedures of the Institutional Ethics Committee of the Public Health Foundation of India and the Emory University Institutional Review Board.

### Data analysis and results of the social network survey

The main CARRS survey, like other health surveys, records basic demographic, socioeconomic, and behavioral information. SNAP therefore did not need to be collected the following respondent information: household size, religion, caste and tribe affiliation (historically marginalized groups), income, sex, age, marital status, whether the person is household head, place of birth, years of education, employment status, years lived at current residence, tobacco and alcohol use, and exercise. Availability of such data offers efficiency to adding a social network instrument to an existing study. CARRS data were extracted and merged with SNAP data using SAS 9.2 (SAS Institute, Cary NC). Preliminary data analysis focused on constructing network measures from the variables, tabulating all variables, checking correlation coefficients.

In our sample, respondents were on average aged 45.4 years (ranging from 20 to 94 years), 51% were male, 75% were Delhi natives, 82% were Hindu, and 38% belonged to a Scheduled Tribe or Caste. 87% were married, 78% were currently employed; 53% had household monthly incomes below Rs 10,000 (dichotomized above vs. below the sample median of 10,001–20,000 rupees/month); they had completed an average of 9.9 years of formal education. In terms of health behaviors, 34% of men were current alcohol drinkers while no women reported alcohol consumption and 39% of men reported current tobacco use compared to 3% of women.

With respect to network characteristics, the average network consisted of 3.8 people (SD = 1.3; 3.9 among women and 3.6 among men, p = 0.03; Table 3); 39% named 5 people and 3% named none. Those who reported no network members were excluded from further analysis. The mean network exposure to tobacco was 8% for women and 15% for men. The measures of network homogeneity, were percent of alters of the same sex as the respondent, of alters with an ego-perceived bodyweight similar to the respondent, and of alters residing in the same city as the respondent. The mean percentage of same-sex network members was 55% for both women and men. For both women and men, the mean percentage of network members residing in the same city as the respondent was over 90%. The mean percentage of network members with bodyweights similar to the respondent was 26% and 36% for men.

**Table 3:** Social network characteristics of adults in New Delhi India.

Network measures	Full Sample	Women	Men	p-value
Sample size of respondents	201	105	103	–
**Network size**				
Number of members named, M (SD)	3.8 (1.3)	3.9 (1.4)	3.6 (1.2)	0.03
Frequency of reported network size, n (%)				<0.01
0 named members	7 (3.4)	4 (3.8)	3 (2.9)	
1	8 (3.9)	3 (2.9)	5 (4.9)	
2	12 (5.8)	8 (7.6)	4 (3.9)	
3	56 (26.9)	18 (17.1)	38 (36.9)	
4	45 (21.6)	19 (18.1)	26 (25.2)	
5 named members	80 (38.5)	53 (50.9)	27 (26.2)	
**Network exposure**				
Percent of network who use tobacco, M (SD)	11.6 (25.7)	8.0 (22.9)	15.3 (27.8)	0.04
**Network homogeneity**				
Percent of network same sex, M (SD)	55.2 (26.5)	55.1 (24.3)	55.3 (28.7)	0.97
Percent of network same bodyweight as self, M (SD)	30.9 (29.4)	25.8 (24.9)	36.0 (32.7)	0.01
Percent of network in same city, M (SD)	90.6 (20.3)	90.0 (21.4)	91.3 (19.2)	0.66
**Network diversity**				
Percent of network family, M (SD)	80.7 (27.2)	84.7 (23.1)	76.7 (30.4)	0.04

Notes: *P*-value based on Chi-square (binary variables) or ANOVA (continuous variables) tests for differences in distribution by gender.

Tie diversity was the percent of a respondent's network were relatives (Table 4). On average 85% of women's alters and 77% of men's alters were family members. Tie composition across all reported networks was computed using as the denominator the 780 reported ties of all 208 respondents. Female relatives (42% of all ties) and male relatives (38% of all ties) were the most frequent tie types reported. Friendship ties were the most frequent non-family ties, with 18% of all ties being friends (24% for men and 14% for women). Among all reported ties irrespective of nomination position, women most often nominated husbands, sons, and daughters, while men most often nominated wives, friends, and brothers.

**Table 4:** Exemplary dyadic features of ties reported by respondents in New Delhi, India.

Dyadic Measures	n (%)	n (%)
**Strongest ties (n = 201 respondents)**	Relative	Non-relative
First named position	180 (90)	21 (10)
Primary contact for health concern	181 (91)	17 (9)
Most frequent snack partner	178 (91)	17 (9)
**Multiplexity of ties (n = 637 relative ties; n = 143 non-relative ties)**		
Ties with whom respondent exercised in past two weeks	51 (8)	24 (17)

Based on all three measures of tie strength, family members were classified as the strongest ties: 89.2% of respondents listed a family member in the first-named position (Table 5). As an intimacy measure, 91% reported that they would turn to a family network member in the event of a health problem. As a frequency of contact measure, 91% named a family member as the most frequent person they shared snacks with in the previous fourteen days.

**Table 5:** Composition of ego-nominated networks by proportion of tie type among all nominated ties.

Tie Type	Full Network (n = 780)	Position 1 (n = 201)	Position 2 (n = 193)	Position 3 (n = 181)	Position 4 (n = 125)	Position 5 (n = 80)
	%	%	%	%	%	%
**Male Relative**	37.7	42.3	34.8	37.8	18.9	14.4
Husband	10.1	28.4	1.6	6.1	4.8	2.5
Son	11.2	4.0	17.6	12.2	9.6	13.8
Son-in-law	0.3	0.0	0.0	0.0	0.8	1.3
Father	2.1	2.0	2.1	2.8	0.8	2.5
Brother	9.0	3.0	10.4	13.3	10.4	8.8
Father-in-law	0.3	0.5	0.5	0.0	0.0	0.0
Brother-in-law	2.7	2.0	2.6	4.4	3.2	0.0
Cousin	1.0	1.0	0.5	1.7	0.0	2.5
Uncle	0.8	1.5	0.5	1.1	0.0	0.0
Nephew	0.4	0.0	0.5	0.6	0.8	0.0
**Female Relative**	42.1	46.8	39.9	38.1	41.6	45
Wife	10.6	30.4	4.2	5.5	1.6	2.5
Daughter	8.5	5.0	9.8	9.9	9.6	8.8
Daughter-in-law	2.2	0.0	1.0	2.8	4.0	6.3
Mother	6.0	5.0	8.8	7.2	3.2	3.8
Sister	6.9	4.5	8.3	6.6	8.8	7.5
Sister-in-law	5.3	2.0	4.2	3.9	11.2	10
Mother-in-law	0.9	0.0	2.6	0.0	0.0	2.5
Cousin	0.9	0.0	0.5	1.7	2.4	0.0
Aunt	0.3	0.0	0.0	0.0	0.0	2.5
Niece	0.5	0.0	0.5	0.6	0.8	1.3
**Non-relative**	18.3	10.4	22.3	18.8	23.2	20
Friend	12.7	8	14.5	13.4	18.4	10
Workmate	1.0	0.5	1.0	0.6	1.6	2.5
Neighbor	4.6	2.0	6.7	5.0	3.2	7.5
**Other**	1.9	0.5	1.6	1.1	4.8	3.8

Tie multiplexity focused on which type of network members engaged with respondents in various activities related to cardiovascular health. Respondents reported 637 ties with relatives and 143 ties with friends, neighbors, or coworkers. Respondents reported that they had engaged in exercise in the past two weeks with 8% of their relative ties and 17% of their friend ties; 78% of respondents reported no exercise partners in the network.

### Steps forward in Understanding Social Networks and Health in LMIC settings

Extensive research from social epidemiology demonstrates the intrinsic connection between social support and individual health. Social networks are important for dissimination of knowledge about health, instrumental support to help promote wellbeing, and sustainibility of behavior change. Considering social networks in health research provides a framework for identifying and quantifying social resources. The sparcity of research on social networks and health in non-LMIC settings limits our understanding of the role of the social context for health. There is an immediate need to expand the use of scientifically guided, empirically tested instruments to efficiently collect basic information about social networks and health. The social network measures introduced in this report provide concrete measurable tools for investigating the relationships between social interactions and health. Researchers can select network measures appropriate for specific testable hypotheses and research purposes.

In this report, we have offered an introduction to the fundamental measures and concepts of social networks for health researchers. We presented lessons learnt from the development and implementation of a survey for garnering a better understanding of the role of social networks in cardio-metabolic health in urban India. This instrument can be used and adapted for use in other settings and easily incorporated as a module in existing health surveys.

Our instrument was designed to collect egocentric data. Egocentric network surveys are easy and low-cost to implement and add minimal burden to respondents. Ego-perceived measures correlate well with actual alter attributes, thus egocentric survey designs can reliably describe true network characteristics [52]. At the same time, for some research questions pertainant to health, sociometric data may be more useful. For example, tracking the diffusion of cigarette smoking throughout a network requires longitudinal, sociometric network meaures. Higher-order network measures such as connectivity, boundedness, centralization, cohesion, and the identification of weak ties also require sociometric data [4], [53]. The notable limitation of egocentric data is the reliance on the respondent to report about his or her relationships and the characterstics of his or her network partners.

Like most other instruments, social network surveys should be conducted with a clear understanding of the population being studied. Even when adapting an existing instrument, it is important to adapt and pretest the instrument in the local context to ensure that the appropriate terminology is used to refer to social network members and activities in a way that will capture the desired concept. Surveys should always be either developed or translated and back-translated, tested, and administered in the local languages.

Furthermore, researchers should note and assess the bias that may be introduced by the local context into the social network instrument, as in to the other components of health surveys. For example, social desirability bias may be of concern as interviews are rarely performed in confidential setting. Reports of health-related behaviors, such as alcohol and smoking, or of social support, may be affected by the presence of other persons during the interview process. Reports may also be affected by the desire to impress or please the interviewer or minimize conflict, given that the research team members may be viewed as person of high authority or social standing and as persons vested in healthy behaviors [51].

Analysis of our data indicated patterns that should be investigated further in urban India and in other settings. Many respondents did not identify 5 network members, which was the predefined upper limit in this survey: adults reported 3.8 ties on average, with only 38% filling all 5 spots available in the survey. Studies in the U.S. also found that all spots on the name generator are generally not filled [54]. Secondly, we found that approximately 80% of ties consisted of family relationships – most often spouses, children, and siblings - with fewer ties reported with friends than we had expected. Third, women were nominated more often than men, and women reported significantly larger social networks than men, consistent with findings of adults in the United States and Australia [55]. Network size and gender composition in our study were consistent with those reported in a social network study of mothers in Southern India, where mean network size was 3.36, 78% of networks were composed of family members, and more female than male ties were nominated among the urban-dwelling sample compared to the rural-dwelling sample [56]. Finally, we found that while the majority (90%) of network ties resided in the same city as the participant, less than 5% of ties were neighbors.

The connections between health and wellbeing among individuals who are socially connected with each other is evidenced and relevant for health research and healthcare practice. Consideration of social ties is imperative in the design of health programs. Social network analysis provides a quantifiable and testable framework to study the impact of social ties on health. This project demonstrated the feasibility and acceptability of a social network survey in a non-Western setting, describing how network instruments can be adapted into existing health surveys and illustrating that relatively few questions can collect a plethora of network measures. Social network analysis can quantify the complex social environment to improve understanding and promotion of health.

## Supporting Information

Material S1 **Social Network Analysis Project (SNAP) instrument.**
(DOCX)

Table S1 **Characteristics of respondents, by gender.**
(DOC)
