# Supplementary material for: Measuring Social Networks for Medical Research in Lower-Income Settings
Source: PLoS One. 2014 Aug 25;9(8):e105161. doi: 10.1371/journal.pone.0105161 (PMC4143257; doi:10.1371/journal.pone.0105161)

| **NO.** | **QUESTION** | **RESPONSE** | | **{A}** | **{B}** | **{C}** | | **{D}** | **{E}** |
| --- | --- | --- | --- | --- | --- | --- | --- | --- | --- |
| S1 | *"Next I would like to ask you a few questions about the closest people in your life. Please think of people with whom you may discuss problems or with whom you would exchange advice. I will ask you for their names to keep track of them for the following questions, but the names will be kept confidential and will not be made available to anyone except the investigators of the study."* | | | | | | | | |
| S2 | Could you please tell me the names of the 5 people whom you consider to be closest to you? They can be family members, friends, acquaintances, or coworkers. Please begin with the person you are closest to. | 1. WRITE THE FIVE NAMES IN APPROPRIATE RESPONSE COLUMN. THE PERSON LISTED FIRST IS {A}, SECOND IS {B}, AND SO ON. 2. IF LESS THAN FIVE ARE NAMED, ASK *"IS THERE ANOTHER PERSON WHO YOU WOULD CONSIDER CLOSE TO YOU? AGAIN THIS PERSON CAN BE A FAMILY MEMBER, FRIEND, COWORKER, OR AQUAINTANCE."* | | ______ | ______ | ______ | | ______ | ______ |
| S3 | Is ___ male or female? READ QUESTION FOR PERSON {A}, AND ASK “HOW ABOUT _____?” FOR {B}-{E}. REPEAT QUESTION IF NECESSARY. | Male……………………….…………………………… 1 Female………………….........................……… 2  (Not applicable…………..……………………... 77) | | ______ | ______ | ______ | | ______ | ______ |
| 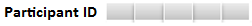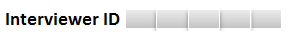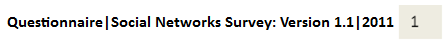S4 | What is _____'s relationship to you?   READ QUESTION FOR PERSON {A}, AND ASK “HOW ABOUT _____?” FOR {B}-{E}. REPEAT QUESTION IF NECESSARY. | Male Relative husband………………………………………….……. 1 son………………………………………………….……. 2 father…………………………………………..………. 3 brother…………………………………………………. 4 father-in-law………………………………..………. 5 brother-in-law…………………………..…………. 6 cousin....................................................... 7 paternal grandfather............................... 8 maternal grandfather.............................. 9 other male relative SPECIFY (______)... 99 Female Relative wife…………………………………………………... 10 daughter……………………………………..……. 11 mother………………………………………..……. 12 sister…………………………………………..……. 13 sister-in-law…………………………………..…. 14 mother-in-law………………………..…………. 15 cousin……………………………………………….. 16 paternal grandmother........................... 17 maternal grandmother.......................... 18 other female relative SPECIFY (______) . 99 Non-relative friend……………………………………………….…. 19 workmate……………………………………..……. 20 neighbor………………………………………….…. 21 other SPECIFY (______)………………………. 99 | | ____ ____ | ____ ____ | ____ ____ | | ____ ____ | ____ ____ |
| **NO.** | **QUESTION** | **RESPONSE** | | **{A}** | **{B}** | **{C}** | | **{D}** | **{E}** |
| S5 | Where does ____ live in relation to you?  **READ SCALE.** READ QUESTION FOR PERSON {A}, AND ASK “HOW ABOUT _____?” FOR {B}-{E}. REPEAT QUESTION AND SCALE IF NECESSARY. | Same household…………………………………... 0 Same building…………………………………….... 1 Same neighborhood……………………………… 2 Same ward………………………………………….… 3 Same city……………………………….……………… 4 Another city or village………………………..…. 5  (Not applicable……………………….………..…. 77) | | ______ | ______ | ______ | | ______ | ______ |
| S6 | Of the 5 people listed, whom would you be most likely to contact if you had a health emergency or if you needed help?  MARK APPROPRIATE CODE FOR CHOSEN PERSON | 1. MARK 1 FOR CHOSEN PERSON 2. IF "DON'T KNOW" OR "REFUSED" MARK FOLLOWING CODE IN {A} BOX  (Not applicable……………………………….…. 77) (Don't know………………………………..…….. 98) (Refused……………………………..…………….. 99) | | ____ ____ | ______ | ______ | | ______ | ______ |
| S7 | Of the 5 people listed, whom would you be most likely to speak with about a health problem?  MARK APPROPRIATE CODE FOR CHOSEN PERSON | 1. MARK 1 FOR CHOSEN PERSON 2. IF "DON'T KNOW" OR "REFUSED" MARK FOLLOWING CODE IN {A} BOX  (Not applicable…………………………….……. 77) (Don't know…………………………………..….. 98) (Refused………………………………..………….. 99) | | ____ ____ | ______ | ______ | | ______ | ______ |
| S8 | Of the 5 people listed, whom would you be most likely to contact if were feeling overwhelmed or anxious?  MARK APPROPRIATE CODE FOR CHOSEN PERSON | 1. MARK 1 FOR CHOSEN PERSON 2. IF "DON'T KNOW" OR "REFUSED" MARK FOLLOWING CODE IN {A} BOX  (Not applicable………………………….………. 77) (Don't know…………………….…….………….. 98) (Refused……………………………….………….. 99) | | ____ ____ | ______ | ______ | | ______ | ______ |
| S9 | How often do you speak to ______ about:  **READ SCALE.** FOR 9a-9b, READ QUESTION FOR PERSON {A}, AND ASK “HOW ABOUT _____?” FOR {B}-{E}. REPEAT QUESTION AND SCALE IF NECESSARY. ASK **HORIZONTALLY** FOR EACH QUESTION, SUCH THAT YOU ASK 9b FOR ALL PERSONS {A}-{E} BEFORE MOVING ON TO 9c. | | |  | | | | | |
| S9a | your own health? | Never……………………………………………………. 1 Very seldom……………………………………….…. 2 Sometimes……………………………………………. 3 Often………………………………………………….…. 4 Regularly………………………………………………. 5 (Not applicable………………………….………. 77) (Don't know…………………………..………….. 98) (Refused……………………..…………………….. 99) | | ____ ____ | ____ ____ | ____ ____ | ____ ____ | | ____ ____ |
| 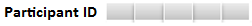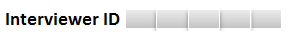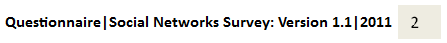S9b | _____'s health? |  |  | ____ ____ | ____ ____ | ____ ____ | ____ ____ | | ____ ____ |
| **NO.** | **QUESTION** | **RESPONSE** | **{A}** | | **{B}** | **{C}** | **{D}** | | **{E}** |
| S10 | *"Now I'm going to ask you some additional questions about activities you have done in the past* ***fourteen*** *days with the people you listed."* | | | | | | | | |
| S11 | Have you communicated with ____ in person, by phone, SMS, or email in the past **fourteen** days?  READ QUESTION FOR PERSON {A}, AND ASK “HOW ABOUT _____?” FOR {B}-{E}. REPEAT QUESTION IF NECESSARY. | No…………………………………………………….….. 0 Yes…………………………………………………….… 1 (Not applicable…………………………………. 77) (Don't know…………………………….……….. 98) (Refused…………………………….…………….. 99) | ____ ____ | | ____ ____ | ____ ____ | ____ ____ | | ____ ____ |
| S12 | In the past **fourteen** days, how many days have you shared snacks with _____?  READ QUESTION FOR PERSON {A}, AND ASK “HOW ABOUT _____?” FOR {B}-{E}. REPEAT QUESTION IF NECESSARY. | NUMBER OF DAYS______  (Not applicable…………………………..……. 77) (Don't know………………………….………….. 98) (Refused…………………………..….………….. 99) | ____ ____ | | ____ ____ | ____ ____ | ____ ____ | | ____ ____ |
| S13 | In the past **fourteen** days, how many days have you shared meals with _____?  READ QUESTION FOR PERSON {A}, AND ASK “HOW ABOUT _____?” FOR {B}-{E}. REPEAT QUESTION IF NECESSARY. | NUMBER OF DAYS______  (Not applicable…………………………………. 77) (Don't know…………………………….……….. 98) (Refused……………………………………….….. 99) | ____ ____ | | ____ ____ | ____ ____ | ____ ____ | | ____ ____ |
| S14 | During the past **fourteen** days, have you done any of the following activities together with _____?  FOR 14a-14e, READ QUESTION FOR PERSON {A}, AND ASK “HOW ABOUT _____?” FOR {B}-{E}. REPEAT QUESTION AND SCALE IF NECESSARY. ASK **HORIZONTALLY** FOR EACH QUESTION, SUCH THAT YOU ASK 9b FOR ALL PERSONS {A}-{E} BEFORE MOVING ON TO 9c. | |  | |  |  |  | |  |
| S14a | Exercised, done yoga, jogged, or gone to the gym with the purpose of maintaining or improving your health? | No…………………………………………………..….. 0 Yes……………………………………………..……… 1 (Not applicable………………………….……. 77) (Don't know……………………………..…….. 98) (Refused………………………………..……….. 99) | ____ ____ | | ____ ____ | ____ ____ | ____ ____ | | ____ ____ |
| S14b | Walked or performed small tasks outside of the home, such as walking to the store? |  | ____ ____ | | ____ ____ | ____ ____ | ____ ____ | | ____ ____ |
| S14c | Prepared a meal together or gone grocery shopping? |  | ____ ____ | | ____ ____ | ____ ____ | ____ ____ | | ____ ____ |
| S14d | Had an alcoholic beverage? |  | ____ ____ | | ____ ____ | ____ ____ | ____ ____ | | ____ ____ |
| 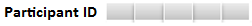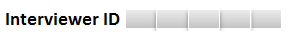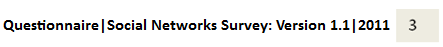S14e | Used tobacco in any form (smoking, chewing, snuff, etc.)? |  | ____ ____ | | ____ ____ | ____ ____ | ____ ____ | | ____ ____ |
| **NO.** | **QUESTION** | **RESPONSE** | **{A}** | | **{B}** | **{C}** | **{D}** | | **{E}** |
| S15 | To the best of your knowledge, does ____ use any form of tobacco (smoking, chewing, snuff, etc.)?  READ QUESTION FOR PERSON {A}, AND ASK “HOW ABOUT _____?” FOR {B}-{E}. REPEAT QUESTION IF NECESSARY. | No……………………………………………………….. 0 Yes………………………………………………….…… 1 (Not applicable…………………………………. 77) (Don't know……………………………….…….. 98) (Refused……………………………….………….. 99) | ____ ____ | | ____ ____ | ____ ____ | ____ ____ | | ____ ____ |
| S16 | How would you describe _____'s weight compared to your own weight?  **READ SCALE.** READ QUESTION FOR PERSON {A}, AND ASK “HOW ABOUT _____?” FOR {B}-{E}. REPEAT QUESTION AND SCALE IF NECESSARY. | Much skinnier………………………………………. 1 Slightly skinnier…………………………….…….. 2 Same…………………………………………………… 3 Slightly fatter…………………………………..……4 Much fatter…………………………………………. 5 (Not applicable…………………………….……. 77) (Don't know……………………………..……….. 98) (Refused…………………………..……………….. 99) | ____ ____ | | ____ ____ | ____ ____ | ____ ____ | | ____ ____ |
| S17 | *"That concludes our interview. Thank you for your time."* | | | | | | | | |
| **COMMENTS FROM INTERVIEWER:**  *These notes should include any point of significance, such as the respondent’s behavior (whether they were open or closed, vague or precise, etc.) or the home environment. PLEASE NOTE who, if any, was present during the interview. Also note any problems or difficultly pertaining to specific questions.* | | | | | | | | | |
|  |  |  |  |  |  |  |  |  |  |
|  |  |  |  |  |  |  |  |  |  |
|  |  |  |  |  |  |  |  |  |  |
|  |  |  |  |  |  |  |  |  |  |
|  |  |  |  |  |  |  |  |  |  |


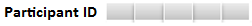

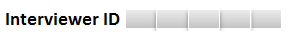

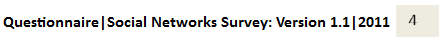

Supplement: Material S1 — Social Network Analysis Project (SNAP) instrument. (DOCX) [file pone.0105161.s001.docx]
