# Supplementary material for: Measuring Social Networks for Medical Research in Lower-Income Settings
Source: PLoS One. 2014 Aug 25;9(8):e105161. doi: 10.1371/journal.pone.0105161 (PMC4143257; doi:10.1371/journal.pone.0105161)
Supplement: Table S1 — Characteristics of respondents, by gender. (DOC) [file pone.0105161.s002.doc]

Appendix 1: Characteristics of respondents, by gender.

| **Characteristics** | **Full Sample** | **Women** | **Men** | **p-value** |
| --- | --- | --- | --- | --- |
| Sample size of respondents | 201 | 105 | 103 |  |
| Age (years), mean (SD) | 45.4 (14.0) | 44.1 (13.2) | 46.6 (14.7) | 0.20 |
| Born in Delhi, n (%) | 75 (37.1) | 35 (34.3) | 40 (40.0) | 0.40 |
| Hindu, n (%) | 169 (81.3) | 88 (83.8) | 81 (78.6) | 0.34 |
| Scheduled tribe or caste, n (%) | 38 (18.6) | 21 (20.6) | 17 (16.7) | 0.47 |
| Currently married, n (%) | 182 (87.5) | 87 (82.9) | 95 (92.2) | 0.04 |
| Employed, n (%) | 88 (42.3) | 8 (7.6) | 80 (77.7) | <.01 |
| Monthly income < Rs 10,001, n (%) | 107 (52.7) | 56 (54.9) | 51 (50.5) | 0.53 |
| Years of education, mean (SD) | 9.9 (5.8) | 8.9 (6.1) | 10.9 (5.3) | 0.01 |
| Current alcohol drinker, n (%) | 35 (16.8) | 0 (0.0) | 35 (34.0) | <.01 |
| Current tobacco user, n (%) | 43 (20.7) | 3 (2.9) | 40 (38.8) | <.01 |

Notes: *P*-value based on Chi-square (binary variables) or ANOVA (continuous variables) tests for differences in distribution by gender.

Source: CARRS Surveillance Study (Nair et al., 2012a).
